# Spatiotemporal patterns and risk mapping of provincial hand, foot, and mouth disease in mainland China, 2014–2017

**DOI:** 10.3389/fpubh.2024.1291361

**Published:** 2024-01-26

**Authors:** Yuxin Wei, Yue Ma, Tao Zhang, Xuelian Luo, Fei Yin, Tiejun Shui

**Affiliations:** ^1^West China School of Public Health and West China Fourth Hospital, Sichuan University, Chengdu, China; ^2^National Institute for Communicable Disease Control and Prevention, Chinese Center for Disease Control and Prevention, Beijing, China; ^3^Yunnan Center for Disease Control and Prevention, Kunming, China

**Keywords:** HFMD, spatiotemporal pattern, Bayesian spatiotemporal model, influencing factor, risk mapping

## Abstract

**Background:**

Hand, foot, and mouth disease (HFMD) has remained a serious public health threat since its first outbreak in China. Analyzing the province-level spatiotemporal distribution of HFMD and mapping the relative risk in mainland China will help determine high-risk provinces and periods of infection outbreaks for use in formulating new priority areas for prevention and control of this disease. Furthermore, our study examined the effect of air pollution on HFMD nationwide, which few studies have done thus far.

**Methods:**

Data were collected on the number of provincial monthly HFMD infections, air pollution, meteorological variables, and socioeconomic variables from 2014 to 2017 in mainland China. We used spatial autocorrelation to determine the aggregate distribution of HFMD incidence. Spatiotemporal patterns of HFMD were analyzed, risk maps were developed using the Bayesian spatiotemporal model, and the impact of potential influencing factors on HFMD was assessed.

**Results:**

In our study, from 2014 to 2017, the HFMD annual incidence rate in all provinces of mainland China ranged from 138.80 to 203.15 per 100,000 people, with an average annual incidence rate of 165.86. The temporal risk of HFMD for 31 Chinese provinces exhibited cyclical and seasonal characteristics. The southern and eastern provinces had the highest spatial relative risk (*RR* > 3) from 2014 to 2017. The HFMD incidence risk in provinces (Hunan, Hubei, and Chongqing) located in central China increased over time. Among the meteorological variables, except for the mean two-minute wind speed (*RR* 0.6878; 95% CI 0.5841, 0.8042), all other variables were risk factors for HFMD. High GDP per capita (*RR* 0.9922; 95% CI 0.9841, 0.9999) was a protective factor against HFMD. The higher the birth rate was (*RR* 1.0657; 95% CI 1.0185, 1.1150), the higher the risk of HFMD. Health workers per 1,000 people (*RR* 1.2010; 95% CI 1.0443, 1.3771) was positively correlated with HFMD.

**Conclusions:**

From 2014 to 2017, the central provinces (Hunan, Hubei, and Chongqing) gradually became high-risk regions for HFMD. The spatiotemporal pattern of HFMD risk may be partially attributed to meteorological and socioeconomic factors. The prevalence of HFMD in the central provinces requires attention, as prevention control efforts should be strengthened there.

## 1 Introduction

Hand, foot, and mouth disease (HFMD) is a widespread infectious illness primarily attributable to enterovirus infection (1). The majority of cases indicate that HFMD is a self-limiting disease. However, a small percentage of patients will have neurological complications and potentially death (2). For more than 20 years, HFMD has occurred frequently in many countries, such as Australia, Malaysia, and China (3). On average, 96 people per 100,000 are infected with HFMD each year during the period from 2008 to 2021 (4). Furthermore, studies have revealed that the average cost per case of HFMD ranges from $990 to $3,000, indicating that HFMD poses a serious economic burden as well (5). As of today, the specific antiviral therapeutic regimen for HFMD remains unavailable. Thus, public health measures related to prevention and control are still important for the management of HFMD.

There have been numerous prior studies on the spatiotemporal pattern of HFMD (6–11). Nevertheless, the majority of the related researches have been carried out in just one province or local region with a small spatial size, such as Shaanxi Province (12), Xinjian (6), Henan Province (13), or the Ili River Valley Region (14). Analysis of the spatial distribution and temporal trend of HFMD incidence nationwide helps to determine high-risk provinces and periods, thus identifying provinces where prevention and control efforts are most needed and maximizing and rationalizing the utilization of limited public health resources. Wang et al. used data from mainland China from 2008 to 2012 to detect spatiotemporal clusters (15). In 2014, the Ministry of Health of China provided new guidance for HFMD prevention and control. Six years have passed since the last relevant policy was issued in 2008. In the first half of 2016, the first inactivated vaccine for EV-A71 was marketed in mainland China (16). The prevalence and spatiotemporal characteristics of national HFMD may be impacted by these interventions to some extent (17–19). However, studies on spatiotemporal patterns that contain fully recorded incidence data on nationwide HFMD cases from 2012 to the present are limited. There was only one study regarding spatiotemporal cluster detection but no risk estimation (20). Therefore, it is necessary to estimate the smoothed map of HFMD risk in recent years to obtain the spatiotemporal risk of HFMD across the country and to observe whether changes have occurred in high-risk provinces.

Identifying risk factors for HFMD is valuable in controlling outbreaks (21). The correlations between HFMD incidence and meteorological factors (22–25), air pollution (26), and socioeconomic factors (27, 28) have been supported by many previous studies. However, most studies on underlying HFMD explanatory variables have concentrated on a specific region or city, and few studies have focused on all of China. In addition, few studies have been conducted to assess the effect of air pollution on the risk of HFMD infection from a national perspective. The impacts of air pollution and meteorological and socioeconomic factors on HFMD have not been explored nationwide. Using Bayesian spatiotemporal models, spatiotemporal variations can be identified simultaneously, the impacts of possible explanatory variables can be assessed (29), and uncertainties due to residual confounding factors can be controlled.

The purpose of the present study was to map the spatiotemporal risk based on a Bayesian spatiotemporal model using province-level HFMD data from 2014 to 2017 in mainland China. The associations between air pollution, meteorological conditions, socioeconomic factors, and HFMD incidence were also analyzed.

## 2 Methods

### 2.1 Study area

**Figure 1:**
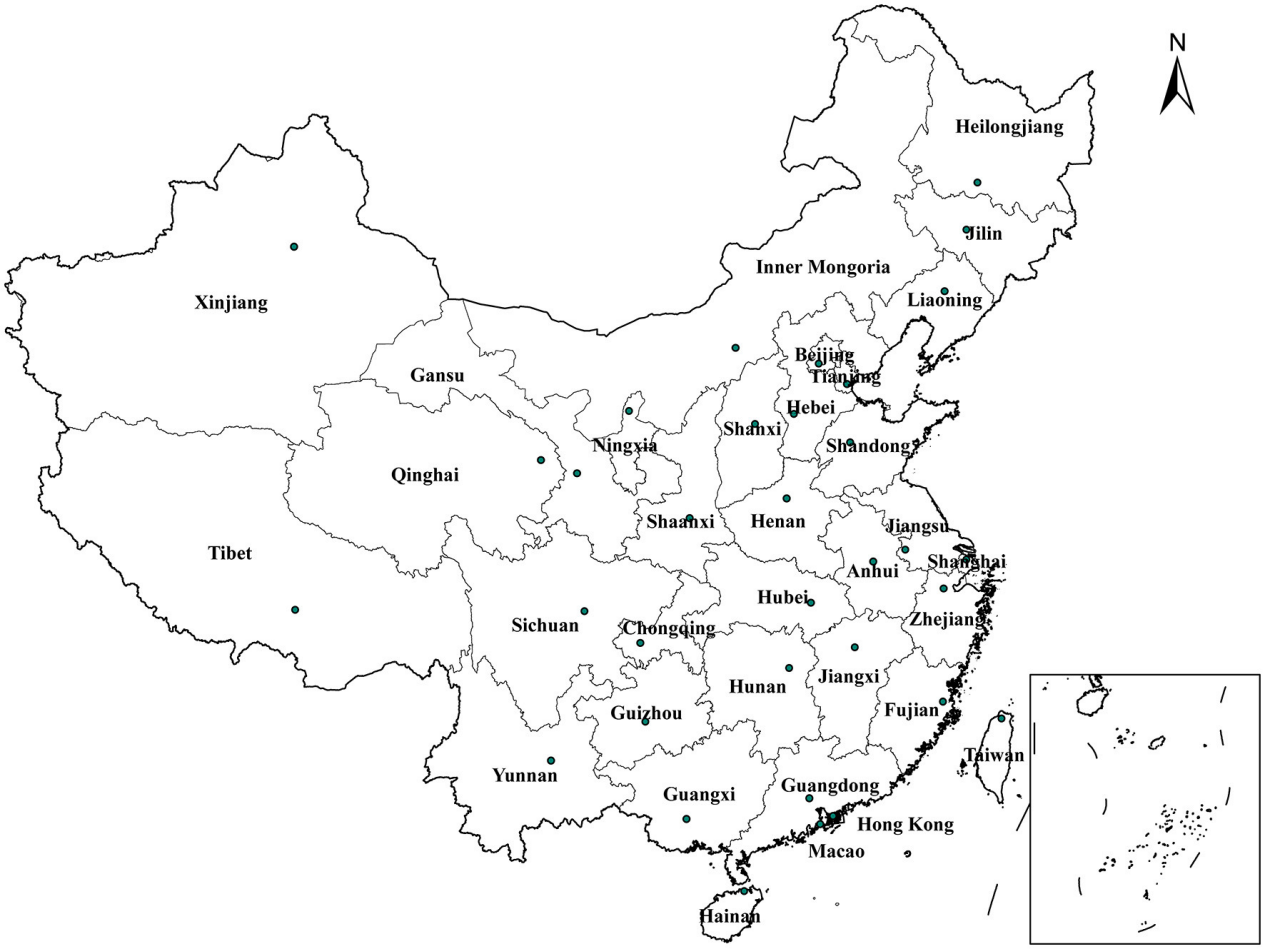
Provincial-level administrative regions in mainland China.

With complex and diverse topographic conditions and vast land area, China is located in the eastern part of Asia. It has several different climate zones, from temperate to subtropical. The topography from the west and east is from high to low. In mainland China, there are 31 provincial administrative regions (Figure 1).

### 2.2 Data sources

We used monthly province-level HFMD case data from January 2014 to December 2017 from China's Public Health Science Data Center (30) and National Health Commission (31).

The raw monthly meteorological data were first collected by us from the China Meteorological Data Service Center (32) for each province's meteorological monitoring stations. First, the location information of the prefecture-level cities in each province was incorporated, and the monthly average data of all the meteorological monitoring stations were used to obtain the monthly average meteorological data of 334 prefecture-level cities in mainland China via the ordinary kriging interpolation method. Subsequently, the annual population of prefecture-level cities within each province was used as a weight to derive the weighted monthly average meteorological data for the corresponding provinces. For the four municipalities, the data from those monitoring sites were averaged to calculate the monthly mean concentrations of meteorological factors. The meteorological variables included the monthly mean temperature (°C), monthly mean rainfall (10 mm), monthly mean relative humidity (%), monthly mean air pressure (hPa), monthly mean hours of sunshine (h), and monthly mean two-minute wind speed (m/s).

Daily air quality indices (AQI) for each prefecture-level city for the same period were collected from the website (33), and monthly statistics for each prefecture-level city were obtained after averaging. Resident population data for each prefecture-level city for each year were incorporated as weights, and a weighted average was used to obtain monthly average AQI statistics for each province (municipality).

Provincial socioeconomic indicators, which were selected from the aspects of the economy, demographic factors, population mobility, and health resources, were obtained from the National Bureau of Statistics (34) and included per capita gross domestic product (GDP_pc_, $ 140.60), health workers per thousand people, hospital beds per 10,000 people, the number of village clinics (10,000), total passenger traffic on public transport (100 million), and birth rate (‰).

### 2.3 Statistical analysis

In this study, spatial autocorrelation and Moran's I were analyzed. Spatial autocorrelation analysis is composed of both global and local indicators. Global spatial autocorrelation analysis mainly studies the spatial distribution of attribute values of regional spatial objects. Whether the distribution of attribute values between two adjacent spatial areas is correlated or random can be obtained through local indicators of spatial association (LISA).

Moran I is computed in Equation 1:


(1) $$I=\frac{n\sum_{i=1}^{n}\sum_{j=1}^{n}\omega_{ij}(y_{i}-\bar{y})(y_{j}-\bar{y})}{\left(\sum_{i=1}^{n}\sum_{j=1}^{n}\omega_{ij}\right){\sum_{i=1}^{n}\left(y_{j}-\bar{y}\right)}^{2}}$$

where *y*_*i*_ or *y*_*j*_ represents the number of HFMD cases in province i or j. The average number of cases in 31 provinces is $\bar{y}$, and ω_*ij*_ denotes the spatial proximity matrix of two provinces, i and j. n is the number of provinces. The larger the absolute Moran I value is, which takes values from −1 to 1, the stronger the correlation. Positive values (*P* < 0.05) indicate positive correlations, and negative values (*P* < 0.05) indicate negative correlations.

LISA is computed in Equation 2:


(2) $$I_{i}=\frac{\sum_{j=1}^{n}\omega_{ij}(y_{i}-\bar{y})(y_{j}-\bar{y})}{\sum_{j=1}^{n}{\left(y_{j}-\bar{y}\right)}^{2}}\;,\;i\neq j$$

*I*_*i*_ = 0 indicates that the distribution of attribute values between adjacent spatial units is random. *I*_*i*_ > 0 indicates that the distribution of attribute values of the spatial object and the neighboring spatial objects has a positive correlation (“high-high” or “low-low”), and *I*_*i*_ < 0 indicates that the distribution has a negative correlation (“high-low” or “low-high”).

Bivariate spatial autocorrelation, usually represented by the bivariate Moran I statistic, characterizes the correlation between one variable and the spatial lag of another. We used the bivariate local indicators of spatial autocorrelation (BiLISA) to capture the relationship between the value of one variable in one spatial unit and the average of another variable in neighboring areas. We drew cluster maps of BiLISA with *P* < 0.05 between meteorological variables, AQI, socioeconomic variables and HFMD incidence.

The BiLISA is computed in Equation 3:


(3) $$\begin{array}{l} BiLISA\;\;\;I_{i}=\frac{\sum_{j=1}^{n}\omega_{ij}\left(x_{i}-\bar{x}\right)\left(y_{j}-\bar{y}\right)}{{\sum_{j=1}^{n}\left(y_{j}-\bar{y}\right)}^{2}}\;,\;i\neq j \end{array}$$

where *x*_*i*_ represents the explanatory variables in province i.

We constructed two Bayesian spatiotemporal models, investigated the fluctuations in HFMD incidence in 31 provinces in mainland China in spatial and temporal dimensions and analyzed the influencing factors. The monthly number of HFMD cases follows a negative binomial distribution, denoted as:


(4) $$\begin{array}{l} y_{it}\sim NegBinomial\left(E_{it}\theta_{it}\right) \end{array}$$

where *E*_*it*_, θ_*it*_ and *y*_*it*_ represent *i* (*i* = 1, 2, …31) provinces *t* (*t* = 1, 2, …48) months of the population, incidence, and the number of cases, respectively.

which is modeled as follows:


(5) $$\begin{array}{l} \eta_{it}=\log\left(\theta_{it}\right)=\beta_{0}+u_{i}+v_{i}+\gamma_{t}+\varphi_{t}+\delta_{it} \end{array}$$

(6) $$\begin{array}{l} \eta_{it}=\log\left(\theta_{it}\right)=\beta_{0}+u_{i}+v_{i}+\gamma_{t}+\varphi_{t}+\delta_{it}+\overset{p}{\underset{k=1}{\sum }}\beta_{k}x_{itk} \end{array}$$

Equation 5 is the model used to estimate the spatial and temporal effects. The intercept β_0_ is equal to the logarithm of the average HFMD incidence in all inland provinces; *u*_*i*_ is the spatially structured random effect, which reflects the spatial autocorrelation among spatial area units; *v*_*i*_ is the spatial heterogeneity; γ_*t*_ is the time structure random effect, taking month as the analysis scale unit, reflecting the overall trend of change in the time effect over the study period; and φ_*t*_ reflects the temporal heterogeneity. δ_*it*_ indicates the interaction effect between space and time.

Equation 6 is the model with potential influencing factors added to Equation 4, where β_*k*_ represents the model coefficient and p represents the number of variables.

β_0_ and β_*k*_ adopted uninformative prior distributions. The prior distribution of *u*_*i*_ is intrinsic conditional autoregression (ICAR) (29), and $v_{i}\sim N\left(0,\sigma_{v}^{2}\right)$. The reciprocal of $\sigma_{u}^{2}$ and $\sigma_{v}^{2}$ as unknown variance parameters are changed into precision parameters τ_*u*_ and τ_*v*_, respectively, and both of their prior distributions are gamma (1, 0.0005). γ_*t*_ follows a second-order autoregressive prior distribution (29). The unstructured time effect follows a normal distribution with a zero mean, $\varphi_{t}\sim N\left(0,\sigma_{\varphi }^{2}\right)$. We assumed the spatiotemporal interaction term to follow a normal distribution with a zero mean; for example, δ_*it*_~*N*(0, τ_δ_).

The preliminary Spearman correlation analysis indicated that rainfall was relatively strongly correlated with the mean temperature (0.78, *P* < 0.001) and mean relative humidity (0.75, *P* < 0.001). Therefore, the initial variable of mean rainfall was removed. Next, the multi-collinearity among other variables was estimated by calculating the variance inflation factor (VIF) indicator. Normally, explanatory variables (VIF > 10) were not included in the Bayesian model due to their severe collinearity. We chose the deviance information criterion (DIC) metric to evaluate the model's goodness-of-fit and select the best Bayesian model. For the same data, the smaller the model DIC value is, the better.

A *P* value < 0.05 indicated that the conclusion was statistically significant. The spatial autocorrelation analyses were conducted using the “spdep” package (R 4.1.2) and GeoDa software version 1.16.0.16. We used ESRI ArcGIS software version 10.8 (Environmental Systems Research Institute, USA) to map the spatial distribution of the data. The Bayesian spatiotemporal models were conducted in R 4.1.2 with the “INLA” package.

## 3 Results

### 3.1 Epidemiological characteristics of HFMD

Table 1 demonstrates the descriptive statistics of the yearly HFMD cases and their incidence in this study. The annual incidence rates varied between 138.80 and 203.15 per 100,000 people. In mainland China, 2014 represented the highest annual incidence of HFMD during the study period, while the lowest number of cases occurred in 2017.

**Table 1:** Annual cases and incidence of HFMD in mainland China from 2014 to 2017.

**Year**	**Case**	**Incidence/100,000**
2014	2,778,861	203.15
2015	1,997,371	145.30
2016	2,442,138	176.61
2017	1,929,550	138.80
Total	9,147,920	165.86

**Figure 2:**
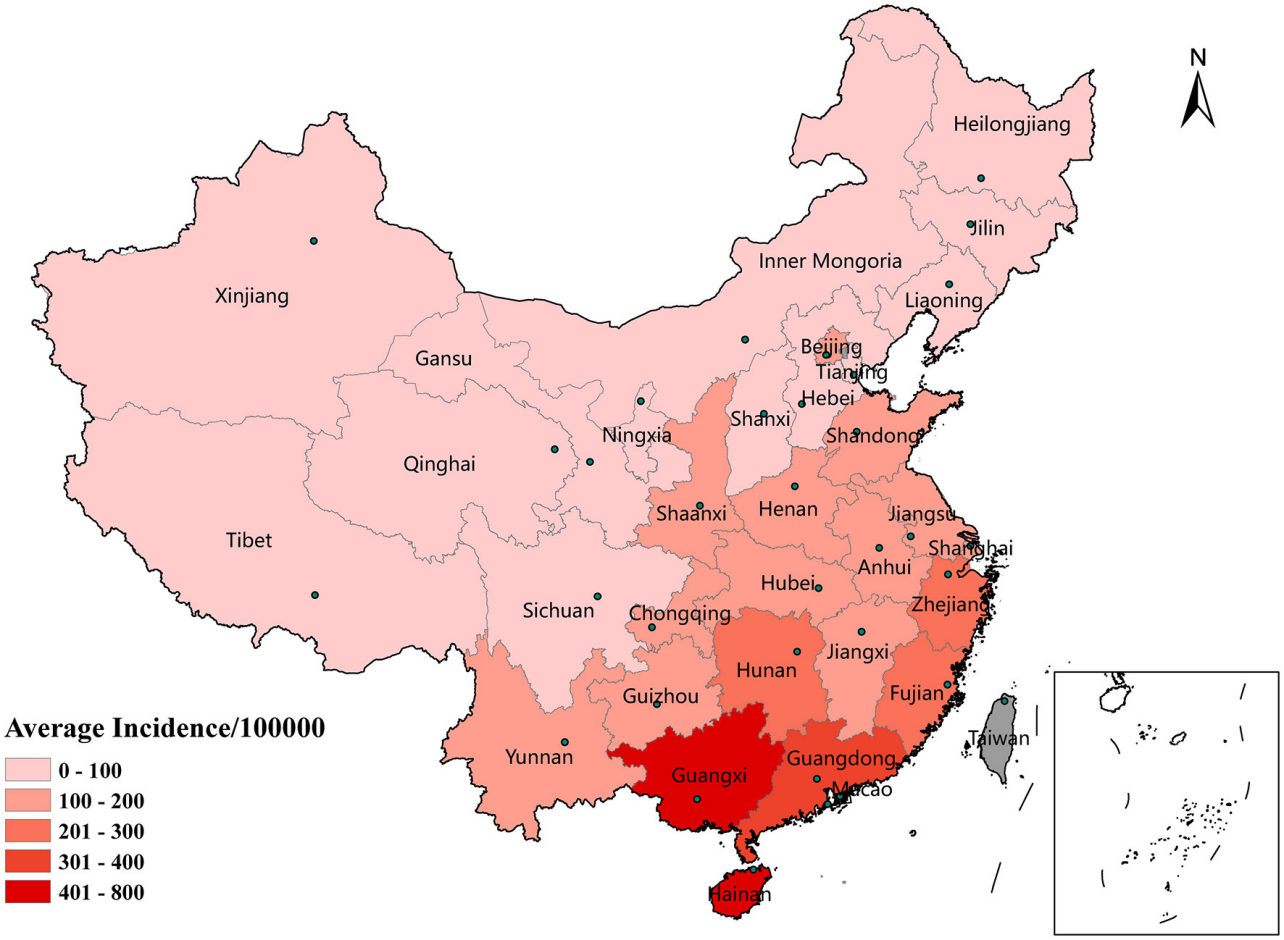
Spatial distribution of the average incidence of HFMD by province in mainland China, 2014–2017.

The spatial distribution of the average annual incidence in each province is shown in Figure 2. The mean incidence of HFMD differed across provinces and varied widely. Guangxi Province had the highest incidence, with an average of 563.01 per 100,000 individuals, followed by Hainan Province (491.97/100,000), Guangdong Province (361.27/100,000), and Fujian Province (242.13/100,000). The incidence of HFMD in the eastern (Jiangsu, Anhui, Fujian, Shanghai, and Shandong Provinces) and southern coastlands (Guangdong, Guangxi, and Hunan Provinces) was higher than that in northwestern (Xinjiang, Qinghai, and Gansu Provinces) and northeastern (Heilongjiang, Jilin, Liaoning, etc.) regions of China.

**Figure 3:**
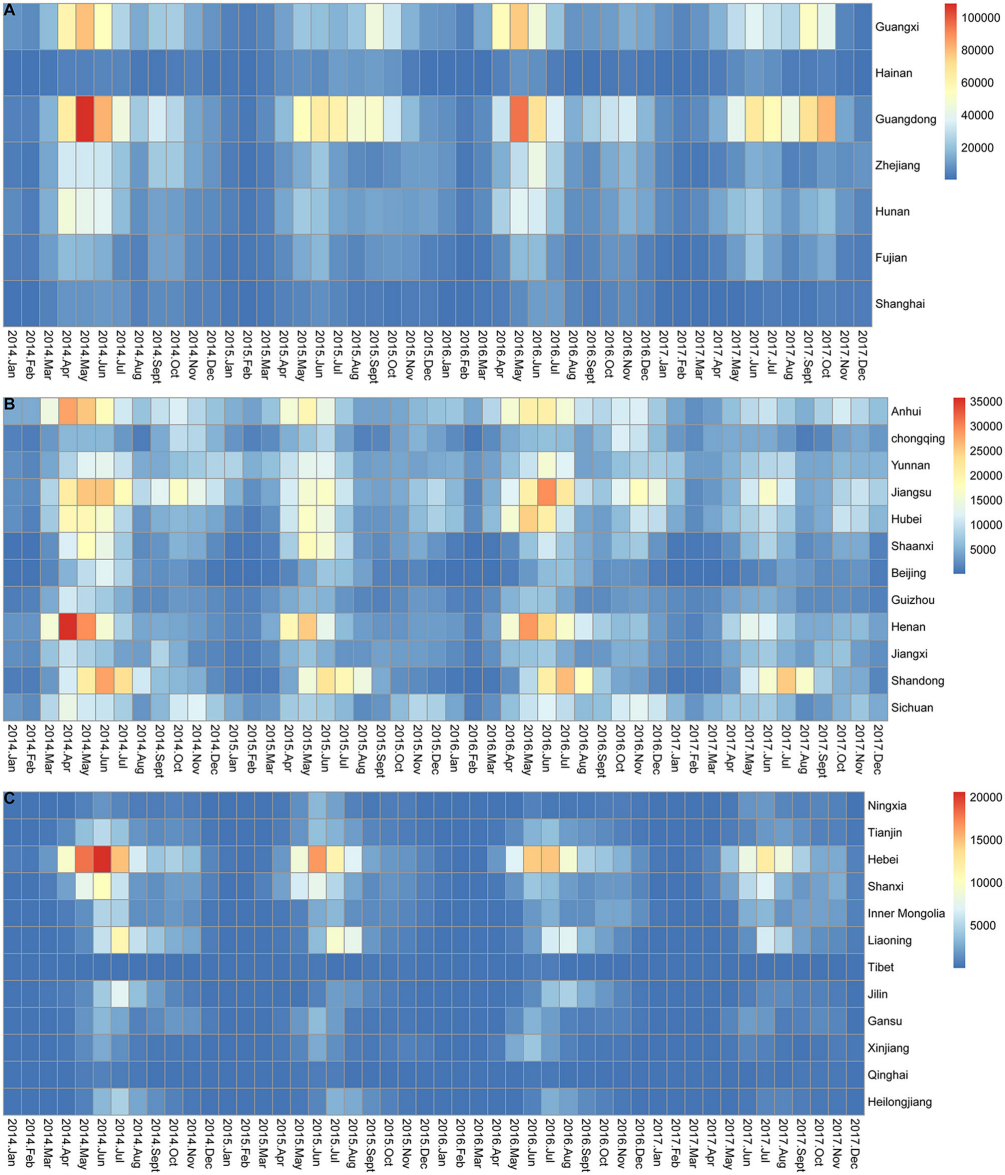
Monthly incidence of HFMD in mainland China from 2014 to 2017. **(A)** Monthly incidence of HFMD in 7 provinces located on the southern and eastern coasts of mainland China. **(B)** Monthly incidence of HFMD in 12 provinces located in central and south-western mainland China. **(C)** Monthly incidence of HFMD in 12 provinces located in western and northern mainland China.

The monthly incidence of HFMD in all provinces generally exhibited seasonal characteristics (Figure 3). As shown in Figures 3A, B, the peak months of HFMD incidence varied in different provinces each year. Provinces with two epidemic peaks each year were located mainly in southern (Guangdong, Guangxi, and Hainan provinces), central (Hunan, Chongqing, and Hubei provinces), and eastern (Jiangsu, Anhui, Fujian, Shanghai, and Shandong provinces) China, with a high peak in May-June and a low peak in September-October. However, as shown in Figure 3C, northern China (e.g., Heilongjiang, Jilin, and Liaoning) had an epidemic peak only in June-July.

### 3.2 Spatial autocorrelation analysis

The global spatial autocorrelation analysis indicated a positive spatial autocorrelation of the provincial HFMD incidence from 2014 to 2017 (Table 2); the Moran I value varied between 0.24 and 0.30, and all the values were statistically significant (*P* < 0.01).

**Table 2:** Global spatial autocorrelation analysis and significance test results.

**Year**	**Moran *I***	***E*(*I*)**	***Var*(*I*)**	***Z*-value**	***P*-value**
2014	0.25	−0.03	0.01	2.48	< 0.01
2015	0.24	−0.03	0.01	2.68	< 0.01
2016	0.30	−0.03	0.01	2.89	< 0.01
2017	0.30	−0.03	0.01	3.00	< 0.01

**Figure 4:**
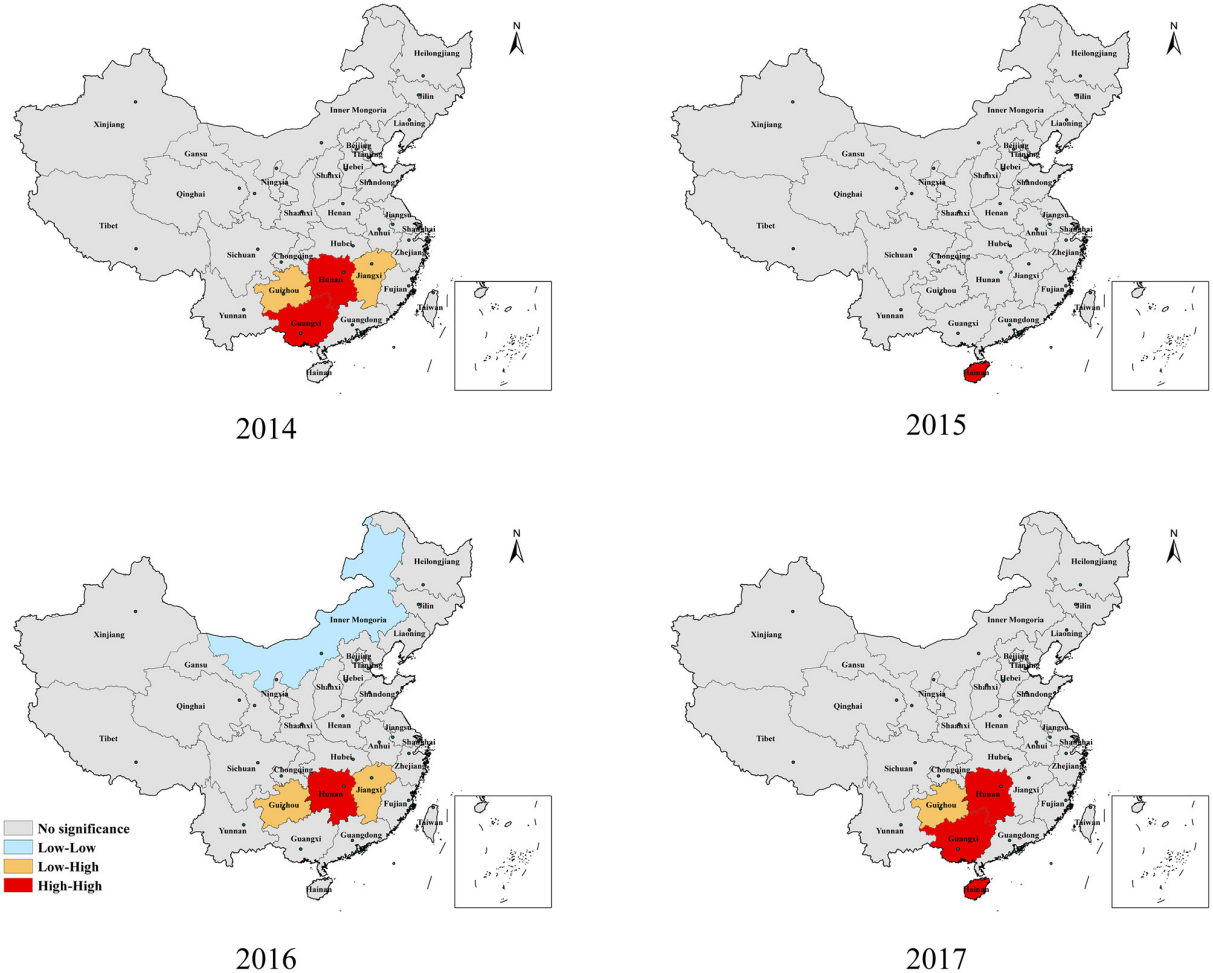
Spatial association cluster map of HFMD incidence in mainland China, 2014–2017.

Figure 4 shows the results of the LISA for each province across mainland China from 2014 to 2017. During the study period, Guangxi, Hainan, and Hunan provinces, located in central and southern China, were the main high-high (HH) regions, and only Hainan Province showed a strong aggregated distribution of HFMD outbreaks in 2015. However, Hunan Province was just the opposite and was the HH province except in 2015. Guizhou and Jiangxi, the two provinces with low-high (LH) clusters, were adjacent to Hunan. Only Inner Mongolia was a low-low (LL) cluster in 2016.

### 3.3 Spatial autocorrelation between variables

**Figure 5:**
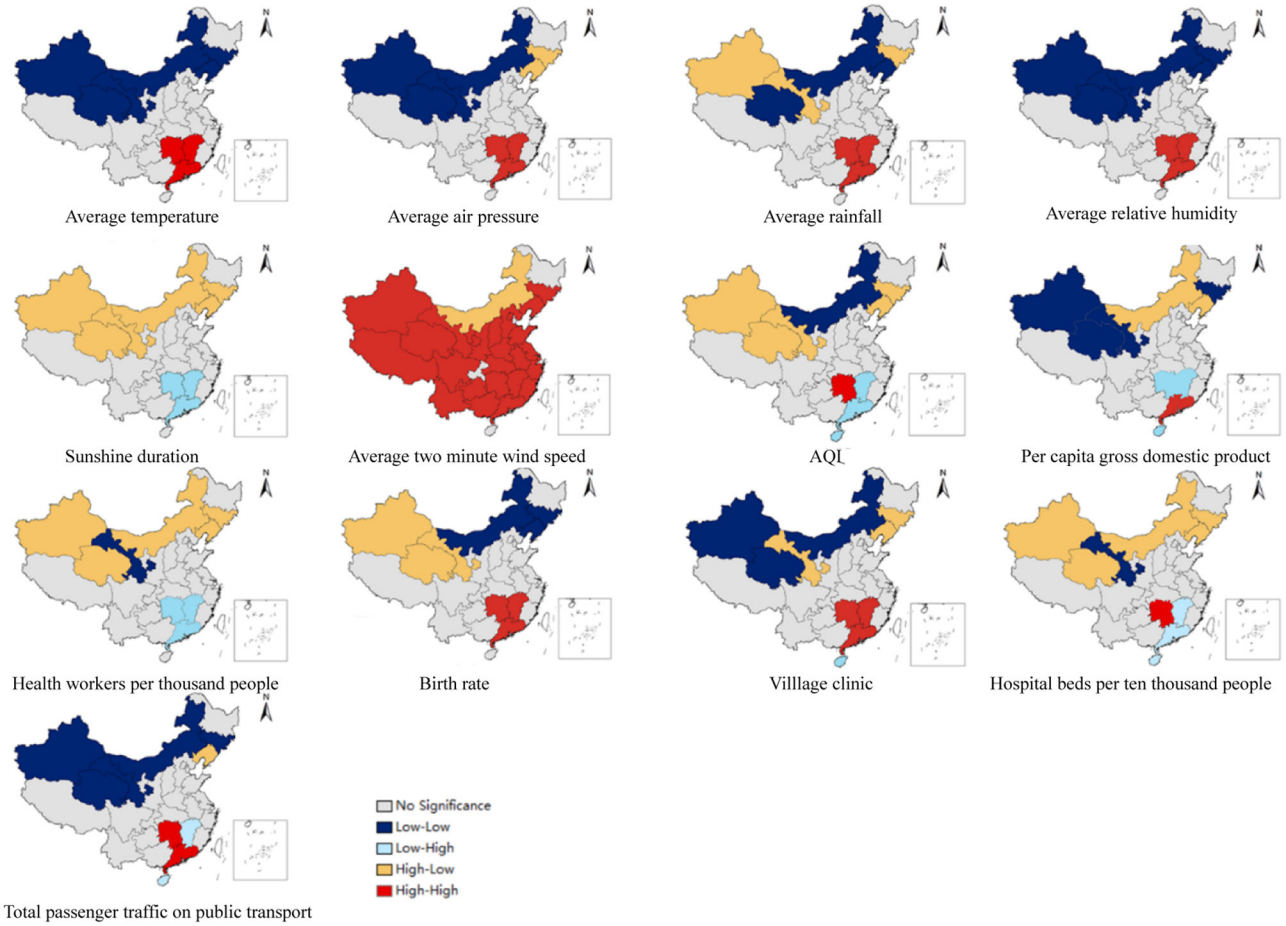
Spatial distribution of the BiLISA between provincial HFMD incidence and influencing factors.

The results of the bivariate local spatial autocorrelation (BiLISA) analysis of the explanatory variables are displayed in Figure 5. Provinces with statistically significant BiLISA values are labeled in different colors, according to the findings, while all other provinces are labeled in gray. Except for the average 2-minute wind speed, we found that the LL and high-low (HL) provinces were situated mostly in Inner Mongolia and northeast and northwest China based on cluster maps of the BiLISA of the relationships between meteorological variables, air pollution, socioeconomic variables, and the incidence of HFMD. This suggests that regardless of whether the meteorological, air pollution and socioeconomic variables of the provinces (e.g., Xinjiang, Inner Mongolia and Jilin) located in these regions were at higher or lower levels, the incidence of HFMD in their neighboring provinces was at lower levels. For the monthly average meteorological variables (except for sunshine hours), the HH provinces were located mainly in central and southern China, where the temperature, rainfall, relative humidity, air pressure, and 2-minute winds in Hunan, Guangdong, and Jiangxi and the incidence of HFMD in neighboring provinces were at high levels. For air pollution and socioeconomic variables (except for health workers per 1,000 people), the HH regions varied by exploratory variables but were still located in south-central China. Especially for Hunan Province, the meteorological variables, air pollution and socioeconomic variables in this region and the incidence of HFMD in neighboring provinces were at high levels.

### 3.4 Spatiotemporal pattern

**Figure 6:**
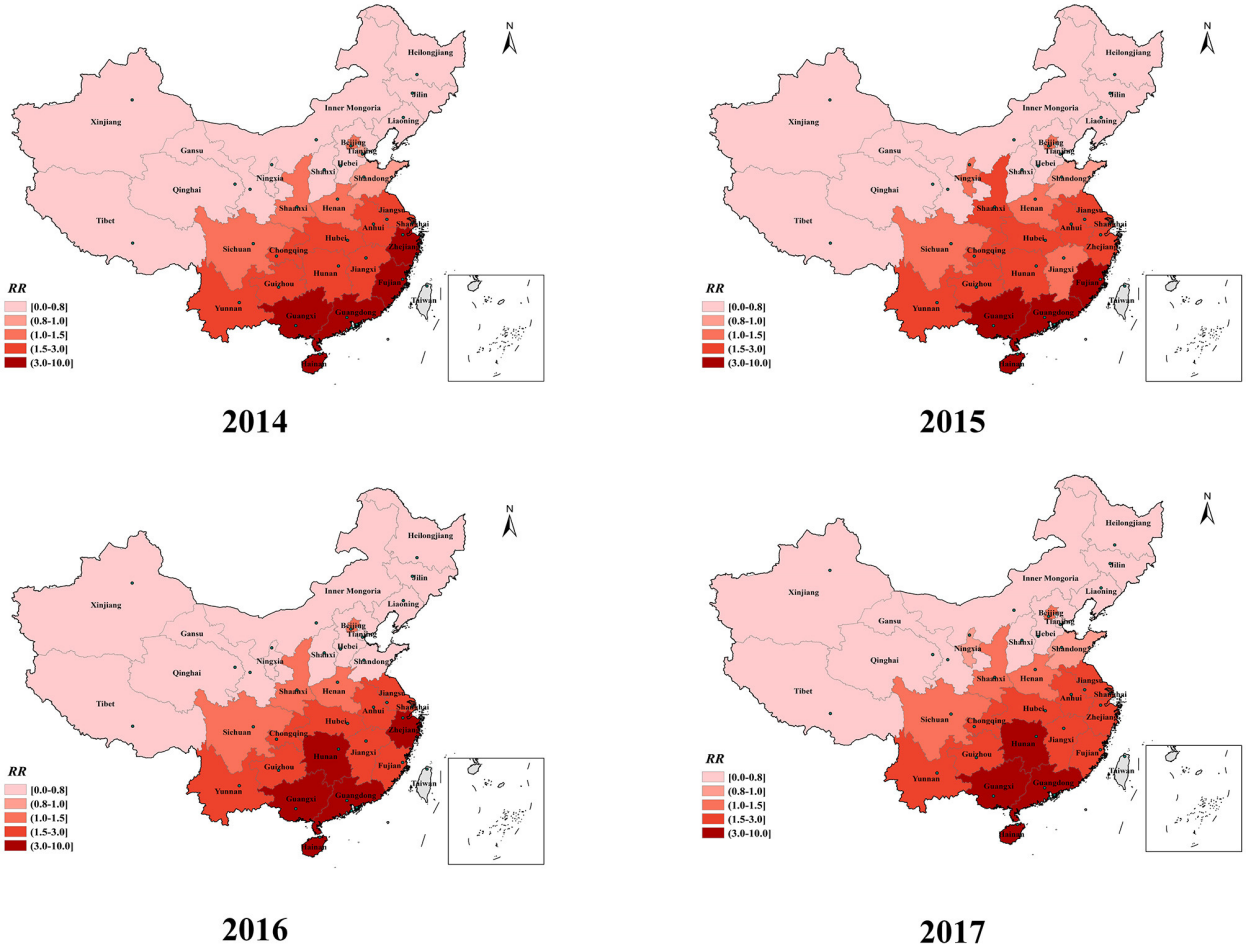
The spatial RR map of HFMD incidence in mainland China from 2014 to 2017.

The spatial relative risk (*RR*) values for HFMD in each of the 31 provinces during the study period are shown in Figure 6. The risk maps showed vast differences in spatial *RR* across provinces. The spatial *RR* (>3.0) was consistently highest in the three southern provinces (Guangdong, Guangxi, and Hainan) shaded in deep red in Figure 6. The spatial *RR* of Hunan Province, located in the central region, gradually increased. The provinces with a low spatial *RR* (< 1.0) were mostly located in the northeast and northwest regions, including Inner Mongolia, Heilongjiang, Jilin, and Tibet Provinces. The provinces located in central and eastern China, including Jiangsu, Anhui, Jiangxi, Chongqing, and Hubei Provinces, had high spatial *RRs* (>1.5). The spatial *RRs* of the two eastern coastal provinces (Fujian and Zhejiang) fluctuated but also remained at a high level.

**Figure 7:**
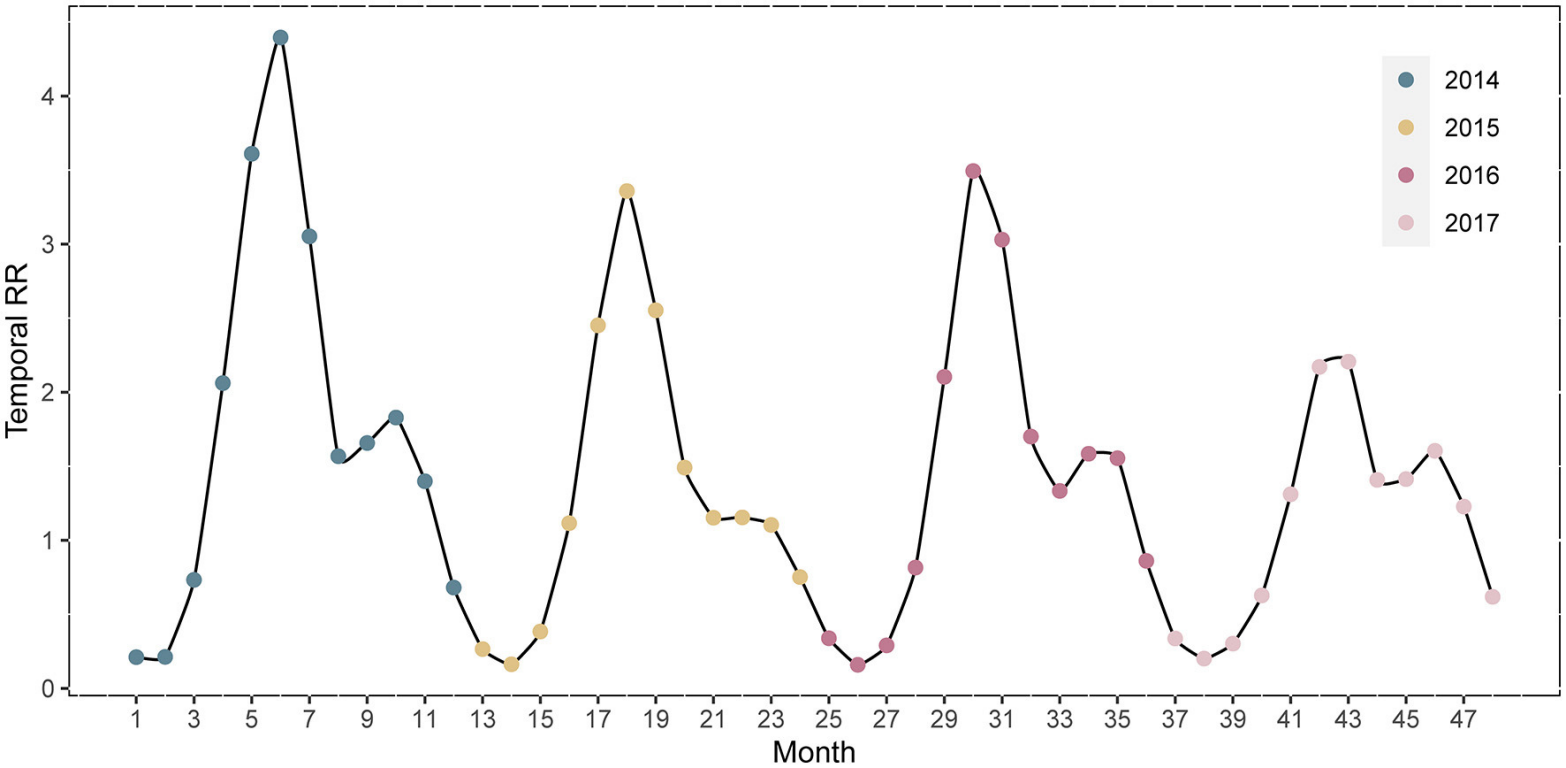
Temporal risk trend of HFMD incidence from 2014 to 2017 in mainland China.

The temporal *RRs* for the 31 Chinese provinces are depicted in Figure 7. The temporal *RR* of HFMD during the study period showed a cyclical pattern and mechanisms of seasonality. HFMD has an epidemic cycle of 2 years. In 2014, the highest temporal *RR* of HFMD occurred from May-June. The difference between the *RRs* of the two peaks in 2017 was the smallest. February was the month with the lowest relative risk.

### 3.5 Explanatory variables on HFMD

The multicollinearity evaluation results are presented in Table 3. All meteorological factors except for the mean 2-minute wind speed were risk factors against HFMD (Table 4). AQI (*RR*: 1.0016; 95% CI: 0.9997, 1.0035) was not significantly associated with the risk of HFMD. Among the socioeconomic factors, GDP_pc_ (*RR*: 0.9922; 95% CI: 0.9841, 0.9999) was negatively correlated with HFMD incidence. In contrast, birth rate (*RR* 1.0657; 95% CI 1.0185, 1.1150) and health workers per 1,000 people (*RR* 1.2010; 95% CI 1.0443, 1.3771) were risk factors of HFMD.

**Table 3:** Multicollinearity evaluation results (VIF, variance inflation factor).

**Variables**	**VIF**	**Variables**	**VIF**
Temperature (°C)	2.582065	Sunshine hours (*h*)	3.146312
Air pressure (hPa)	3.075657	AQI	2.039024
Two-minute wind speed (m/s)	1.966716	GDP_pc_ ($ 140.60)	3.254214
Relative humidity (%)	5.561087	Health workers per thousand people	2.195563
Birth rate (‰)	1.694786	Village clinics (10,000)	1.773240
Total passenger traffic on public transport (100 million)	1.993718	Hospital beds per 10,000 people	1.377031

**Table 4:** Posterior distribution of intercept terms and influencing factors.

**Variables**	**Coefficients**		***RR***	
	**Mean**	**95% CI**	**Mean**	**95% CI**
(Intercept)	−8.7919	(−12.3206, −5.6346)^*^	0.0006	(0.0000, 0.0035)^*^
**Meteorological factors**				
Monthly mean temperature (*°C*)	0.0993	(0.0885, 0.1100)^*^	1.1044	(1.0925, 1.1162)^*^
Monthly mean air pressure (*hPa*)	0.0030	(0.0002, 0.0061)^*^	1.0030	(1.0002, 1.0061)^*^
Monthly mean 2–minute wind speed (*m/s*)	−0.3776	(−0.5386, −0.2169)^*^	0.6878	(0.5841, 0.8042)^*^
Monthly mean relative humidity (%)	0.0402	(0.0324, 0.0479)^*^	1.0410	(1.0330, 1.0490)^*^
Monthly mean sunshine hours (*h*)	0.0035	(0.0022, 0.0048)^*^	1.0035	(1.0022, 1.0048)^*^
**Air pollution**				
AQI	0.0016	(−0.0004, 0.0035)	1.0016	(0.9997, 1.0035)
**Socioeconomic factors**				
GDP_pc_ ($ 140.60)	−0.0079	(−0.0161, 0.0000)^*^	0.9922	(0.9841, 0.9999)^*^
Health workers per 1,000 people	0.1806	(0.0427, 0.3208)^*^	1.2010	(1.0443, 1.3771)^*^
Birth rate (‰)	0.0633	(0.0181, 0.1091)^*^	1.0657	(1.0185, 1.1150)^*^
Hospital beds per 10,000 people	−0.0025	(−0.0243, 0.0207)	0.9975	(0.9761, 1.0207)
Total passenger traffic on public transport (100 million)	0.0004	(−0.0091, 0.0099)	1.0004	(0.9910, 1.0099)
The number of village clinics (10,000)	−0.1036	(−0.2327, 0.0151)	0.9034	(0.7929, 1.0144)

^*^p < 0.05.

## 4 Discussion

Obtaining HFMD risk across the Chinese mainland is of great value for the overall allocation and optimization of public health resources. However, studies of national-scale spatiotemporal analyses of HFMD data in China are limited. In this study, we focused on characterizing the global spatiotemporal pattern of HFMD risk and producing a provincial risk map for the Chinese mainland from 2014 to 2017. Our study is the first to explore the association between AQI and HFMD on a national scale. During the study period, southern Chinese provinces had a stable high risk of HFMD, while the risk in eastern provinces fluctuated. From 2014 to 2017, the central provinces (Hunan, Hubei, and Chongqing) gradually became high-risk areas. Meteorological and socioeconomic variables partially explain this spatiotemporal pattern.

The temporal relative risk of HFMD from 2014 to 2017 indicated the cyclical and seasonal characteristics of the disease in mainland China. HFMD had an epidemic cycle of 2 years. There was evidence that the decline in population immunity between adjacent outbreaks and the increase in the number of newborns led to this cyclical pattern (35). The epidemic of HFMD in mainland China was characterized by two seasonal peaks at the beginning of summer and autumn, with the former being strong and the latter weak, which was consistent with the findings previous research (2). The seasonal prevalence in southern and northern mainland China, however, was inconsistent. We found that only one epidemic peak in northern China occurred in June-July. The onset of HFMD was affected by latitude to some extent according to previous studies (36); for example, tropical and temperate regions located at low latitudes had a higher incidence of HFMD. The duration of the epidemic periodicity of HFMD varies with latitude, with southern regions located at low latitudes showing the strongest semiannual periodicity, but northern regions at high latitudes showing a 1-year periodicity (1). Furthermore, meteorological factors and socioeconomic conditions in northern and southern China showed great discrepancies due to different geographical topography and development, and regional studies also showed different epidemiological patterns of HFMD (37). This explains the discrepancy in the seasonal characteristics of HFMD between northern and southern China.

The spatial *RR* of provinces located in the eastern coastlands remained at a high level (ranging from 1.5 to 10) during the study period in the spatial dimension. However, from 2014 to 2017, high-risk provinces in China were gradually concentrated in the southern and central parts of the country. In our study, three coastal provinces in southern China, namely Guangdong, Guangxi, and Hainan, consistently exhibited the highest risk (>3.0) of HFMD. The predominant climate types in the eastern and southern coastal provinces are subtropical and tropical monsoon climates. These provinces, which have adequate rainfall, heat, and humidity, are more likely to experience HFMD outbreaks (38–40). The high population density and mobility of people in coastal provinces, with many opportunities for interpersonal communication and contact, promote the transmission of the virus, resulting in a higher risk of HFMD spread (37, 41, 42). Moreover, the spatial *RR* map revealed that the central provinces of China have gradually become high-risk regions for HFMD. Driven by the country's macroeconomic policies, since the time that the central China rise strategy was proposed, plenty of supportive policies have been given to urbanization, infrastructure development and transportation network improvement in the central regions, which has led to high levels of migration (43). Furthermore, the central provinces have abundant heat, sunshine duration and high humidity because of short, mild winters and long summers. A suitable natural environment and socioeconomic conditions contribute to the spread and reproduction of the virus in the central region (44, 45). These findings indicated that the epidemic of HFMD in the central provinces is becoming more serious and that prevention and control efforts should be strengthened.

This study suggested that temperature, air pressure, relative humidity, and sunlight duration all increase the risk of HFMD, which was consistent with previous studies (46, 47). Hot and humid environments contributed to the breeding of enteroviruses (48, 49). Higher wind speed and HFMD risk had a negative correlation. Higher wind speeds may accelerate evaporation, making it difficult for the virus to multiply and survive in dry food and environments (50, 51). The infection rate of certain airborne diseases decreased as ventilation rates in the environment increased according to the results of a systematic review (52). Hence, high ventilation resulting from high wind speed reduces the opportunities for HFMD. Air pollution (AQI) was not related to the risk of contracting HFMD. The risk of HFMD decreased with better economic conditions (GDP_pc_, *RR*: 0.9889), which was consistent with previous studies (53). The higher the per capita GDP of the region is, the larger the budget for disease prevention and control. The infected individuals can be treated in time, and the less likely it is that other uninfected children will contract HFMD. Birth rate (*RR* = 1.2010) was a risk factor for HFMD incidence. In particular, health workers per 1,000 people (*RR* 1.2010; 95% CI 1.0443, 1.3771) increased the risk of HFMD. Areas with more healthcare workers tend to have more healthcare resources and better healthcare systems. Medical facilities can report disease immediately to higher health authorities, resulting in a tendency toward higher risk of morbidity (54).

In this study, bivariate local spatial autocorrelation was utilized to investigate the relationships between the explanatory variables of each province and the HFMD incidence in neighboring provinces. The analysis showed a polarization trend, with LL and HH provinces located in northwestern and southern China, respectively. The burden of HFMD incidence in southern China (Guangxi, Hainan) as well as in provinces along the eastern coast of China (Fujian, Zhejiang) and the monthly meteorological variables (including temperature, rainfall, relative humidity, barometric pressure, and 2-minute wind speed) in south-central China were high levels, which can be attributed to the fact that these areas were all in the same climatic zone of China and had similar climatic conditions. The socioeconomic variables of Guangdong, Jiangxi, and Hunan and the incidence of HFMD in surrounding provinces were at high levels, which may partially explain the emergence and persistence of provinces at high risk of HFMD in and around south-central China. Moreover, Hunan Province, a HH region, provides further evidence of the gradual transformation of the central region into a high-risk area for HFMD.

Several limitations existed in our study. First, the meteorological data we used may not be consistent with the climatic conditions of HFMD patients at the time of infection, as these data were obtained from meteorological monitoring stations in each province and could not be specific to individuals. Secondly, some mild cases of HFMD were underreported because infected individuals did not always go to the hospital, resulting in surveillance systems not recording the actual number of HFMD patients.

## 5 Conclusion

Within the present study, we analyzed spatiotemporal patterns using national provincial HFMD data from 2014 to 2017, estimated spatial smoothing maps of risk, and explored potential influencing factors. The risk of HFMD was characterized by cyclical and seasonal patterns in mainland China. The central provinces (Hunan, Hubei, and Chongqing) gradually became high-risk areas for HFMD during 2014–2017. Prevention and control efforts should be strengthened in the central region of China.

## Data availability statement

The original contributions presented in the study are included in the article/supplementary material, further inquiries can be directed to the corresponding authors.

## Ethics statement

This study was carried out in accordance with relevant guidelines and regulations and approved by the Institutional Review Board of the School of Public Health, Sichuan University. The monthly HFMD data used in this current study was aggregated at the province level by counts, therefore no confidential information was used. Written informed consent was not required in accordance with the national legislation and the institutional requirements.

## Author contributions

YW: Writing—original draft. YM: Writing—original draft, Writing—review & editing. TZ: Writing—review & editing. XL: Writing—original draft. FY: Writing—original draft, Writing—review & editing. TS: Writing—review & editing.

## References

[1] Xing W Liao Q Viboud C Zhang J Sun J Wu JT. Hand, foot, and mouth disease in China, 2008-12: an epidemiological study. Lancet Infect Dis. (2014) 14:308–18. 10.1016/S1473-3099(13)70342-6 24485991 PMC4035015

[2] Zhuang ZC Kou ZQ Bai YJ Cong X Wang LH Li C. Epidemiological research on hand, foot, and mouth disease in mainland China. Viruses. (2015) 7:6400–11. 10.3390/v7122947 26690202 PMC4690870

[3] McMinn P Lindsay K Perera D Chan HM Chan KP Cardosa MJ. Phylogenetic analysis of enterovirus 71 strains isolated during linked epidemics in Malaysia, Singapore, and Western Australia. J Virol. (2001) 75:7732–8. 10.1128/JVI.75.16.7732-7738.2001 11462047 PMC115010

[4] BMICC. National Scientific Data Sharing Platform for Population and Health. (2021). Available online at: http://www.bmicc.cn/web/share/home (accessed June 13, 2021).

[5] Zheng Y Jit M Wu JT Yang J Leung K Liao Q. Economic costs and health-related quality of life for hand, foot and mouth disease (HFMD) patients in China. PLoS ONE. (2017) 12:e0184266. 10.1371/journal.pone.0184266 28934232 PMC5608208

[6] Sun S Li Z Hu X Huang R. Spatiotemporal characters and influence factors of hand, foot and mouth epidemic in Xinjiang, China. PLoS ONE. (2021) 16:e0254223. 10.1371/journal.pone.0254223 34428212 PMC8384200

[7] Liu J Xiang X Pu Z Long Y Xiao D Zhang W. Epidemic pattern of hand-foot-and-mouth disease in Xi'an, China from 2008 through 2015. BMC Infect Dis. (2019) 19:19. 10.1186/s12879-018-3624-5 30616531 PMC6322279

[8] Huang R Wei J Li Z Gao Z Mahe M Cao W. Spatial-temporal mapping and risk factors for hand foot and mouth disease in northwestern inland China. PLoS Negl Trop Dis. (2021) 15:e0009210. 10.1371/journal.pntd.0009210 33760827 PMC8021183

[9] Liu W Ji H Shan J Bao J Sun Y Li J. Spatiotemporal dynamics of hand-foot-mouth disease and its relationship with meteorological factors in Jiangsu Province, China. PLoS ONE. (2015) 10:e0131311. 10.1145/2818302 26121573 PMC4488144

[10] Shi RX Wang JF Xu CD Lai SJ Yang WZ. Spatiotemporal pattern of hand-foot-mouth disease in China: an analysis of empirical orthogonal functions. Public Health. (2014) 128:367–75. 10.1016/j.puhe.2014.01.005 24726412

[11] Liu L Zhao X Yin F Lv Q. Spatio-temporal clustering of hand, foot and mouth disease at the county level in Sichuan province, China, 2008-2013. Epidemiol Infect. (2015) 143:831–8. 10.1017/S0950268814001587 25703402 PMC9507101

[12] Ding L Zhang N Zhu B Liu J Wang X Liu F. Spatiotemporal characteristics and meteorological determinants of hand, foot and mouth disease in Shaanxi Province, China: a county-level analysis. BMC Public Health. (2021) 21:374. 10.1186/s12889-021-10385-9 33596869 PMC7890844

[13] Xu C Zhang X Xiao G. Spatiotemporal decomposition and risk determinants of hand, foot and mouth disease in Henan, China. Sci Total Environ. (2019) 657:509–16. 10.1016/j.scitotenv.2018.12.039 30550914

[14] Yi S Wang H Yang S Xie L Gao Y Ma C. Spatial and temporal characteristics of hand-foot-and-mouth disease and its response to climate factors in the Ili river valley region of China. Int J Environ Res Public Health. (2021) 18:41954. 10.3390/ijerph18041954 33671423 PMC7923010

[15] Wang C Li X Zhang Y Xu Q Huang F Cao K. Spatiotemporal cluster patterns of hand, foot, and mouth disease at the county level in Mainland China, 2008-2012. PLoS ONE. (2016) 11:e0147532. 10.1371/journal.pone.0147532 26809151 PMC4726594

[16] Chinese Centre for Disease Control Prevention. Technical Guideline on Use of Inactivated Enterovirus 71 Vaccine. (2016). Available online at: http://www.chinacdc.cn/zxdt/201606/W020160608725047001222.pdf (accessed December 12, 2023).

[17] Yang B Liu F Liao Q Wu P Chang Z Huang J. Epidemiology of hand, foot and mouth disease in China, 2008 to 2015 prior to the introduction of EV-A71 vaccine. Euro Surveill. (2017) 22:824. 10.2807/1560-7917.ES.2017.22.50.16-00824 29258646 PMC5743100

[18] Wang J Jiang L Zhang C He W Tan Y Ning C. The changes in the epidemiology of hand, foot, and mouth disease after the introduction of the EV-A71 vaccine. Vaccine. (2021) 39:3319–23. 10.1016/j.vaccine.2021.05.009 33994239

[19] Jiang H Zhang Z Rao Q Wang X Wang M Du T. The epidemiological characteristics of enterovirus infection before and after the use of enterovirus 71 inactivated vaccine in Kunming, China. Emerg Microbes Infect. (2021) 10:619–28. 10.1080/22221751.2021.1899772 33682641 PMC8018479

[20] Wu Y Wang T Zhao M Dong S Wang S Shi J. Spatiotemporal cluster patterns of hand, foot, and mouth disease at the province level in mainland China, 2011-2018. PLoS ONE. (2022) 17:e0270061. 10.1371/journal.pone.0270061 35994464 PMC9394824

[21] World Health Organization Regional Office for the Western Pacific. A Guide to Clinical Management and Public Health Response for Hand, Foot and Mouth Disease (HFMD). WHO Regional Office for the Western Pacific (2011). Available online at: https://iris.who.int/handle/10665/207490

[22] Xu MM Su T Liu YY Zhao WN Yu QL Qi SX. Analysis on influence and lag effects of meteorological factors on incidence of hand, foot and mouth disease in Shijiazhuang, 2017-2019. Zhonghua Liu Xing Bing Xue Za Zhi. (2021) 42:827–32. 10.3760/cma.j.cn112338-20200930-01213 34814474

[23] Jiang Y Xu J Lai H Lin H. Association between meteorological parameters and hand, foot and mouth disease in Mainland China: a systematic review and meta-analysis. Iran J Public Health. (2021) 50:1757–65. 10.18502/ijph.v50i9.7046 34722370 PMC8542837

[24] Pearson D Basu R Wu XM Ebisu K. Temperature and hand, foot and mouth disease in California: an exploratory analysis of emergency department visits by season, 2005-2013. Environ Res. (2020) 185:109461. 10.1016/j.envres.2020.109461 32278924

[25] Qi H Li Y Zhang J Chen Y Guo Y Xiao S. Quantifying the risk of hand, foot, and mouth disease (HFMD) attributable to meteorological factors in East China: a time series modelling study. Sci Total Environ. (2020) 728:138548. 10.1016/j.scitotenv.2020.138548 32361359

[26] Huang R Ning H He T Bian G Hu J Xu G. Impact of PM(10) and meteorological factors on the incidence of hand, foot, and mouth disease in female children in Ningbo, China: a spatiotemporal and time-series study. Environ Sci Pollut Res Int. (2019) 26:17974–85. 10.1007/s11356-018-2619-5 29961907

[27] Bo YC Song C Wang JF Li XW. Using an autologistic regression model to identify spatial risk factors and spatial risk patterns of hand, foot and mouth disease (HFMD) in Mainland China. BMC Public Health. (2014) 14:358. 10.1186/1471-2458-14-358 24731248 PMC4022446

[28] Huang J Wang J Bo Y Xu C Hu M Huang D. Identification of health risks of hand, foot and mouth disease in China using the geographical detector technique. Int J Environ Res Public Health. (2014) 11:3407–23. 10.3390/ijerph110303407 24662999 PMC3987041

[29] Cameletti M Blangiardo M. Spatial and Spatio-Temporal Bayesian Models With R-INLA. John Wiley & Sons (2015). p. 179.

[30] Phsciencedata. The Data-center of China Public Health Science. (2021). Available online at: http://www.phsciencedata.cn/ (accessed June 13, 2021).

[31] NHC. National Health Commission of the People's Republic of China. (2021). Available online at: http://www.nhc.gov.cn/ (accessed June 13, 2021).

[32] CMA. China Meteorological Data Service Centre. (2021). Available online at: http://data.cma.cn/ (accessed June 13, 2021).

[33] Aqistudy. Air Quality Online Monitoring Analysis Platform. (2021). Available online at: https://www.aqistudy.cn/ (accessed June 13, 2021).

[34] STATS. National Bureau of Statistics of China. (2021). Available online at: http://www.stats.gov.cn/ (accessed June 13, 2021).

[35] NikNadia N Sam IC Rampal S WanNorAmalina W NurAtifah G Verasahib K. Cyclical patterns of hand, foot and mouth disease caused by enterovirus a71 in Malaysia. PLoS Negl Trop Dis. (2016) 10:e0004562. 10.1371/journal.pntd.0004562 27010319 PMC4806993

[36] Tang JH Chan TC Shigematsu M Hwang JS. Latitude-based approach for detecting aberrations of hand, foot, and mouth disease epidemics. BMC Med Inform Decis Mak. (2015) 15:113. 10.1186/s12911-015-0236-5 26703896 PMC4691017

[37] Chen B Yang Y Xu X Zhao H Li Y Yin S. Epidemiological characteristics of hand, foot, and mouth disease in China: a meta-analysis. Medicine. (2021) 100:e25930. 10.1097/MD.0000000000025930 34011066 PMC8137076

[38] Zhang W Du Z Zhang D Yu S Hao Y. Quantifying the adverse effect of excessive heat on children: an elevated risk of hand, foot and mouth disease in hot days. Sci Total Environ. (2016) 541:194–9. 10.1016/j.scitotenv.2015.09.089 26409149

[39] Cheng Q Bai L Zhang Y Zhang H Wang S Xie M. Ambient temperature, humidity and hand, foot, and mouth disease: a systematic review and meta-analysis. Sci Total Environ. (2018) 625:828–36. 10.1016/j.scitotenv.2018.01.006 29306826

[40] Rui J Luo K Chen Q Zhang D Zhao Q Zhang Y. Early warning of hand, foot, and mouth disease transmission: a modeling study in mainland, China. PLoS Negl Trop Dis. (2021) 15:e0009233. 10.1371/journal.pntd.0009233 33760810 PMC8021164

[41] Deng T Huang Y Yu S Gu J Huang C Xiao G. Spatial-temporal clusters and risk factors of hand, foot, and mouth disease at the district level in Guangdong Province, China. PLoS ONE. (2013) 8:e56943. 10.1371/journal.pone.0056943 23437278 PMC3578924

[42] Huang JX Wang JF Li ZJ Wang Y Lai SJ Yang WZ. Visualized exploratory spatiotemporal analysis of hand-foot-mouth disease in Southern China. PLoS ONE. (2015) 10:e0143411. 10.1371/journal.pone.0143411 26605919 PMC4659604

[43] Wu R Li Z Wang S. The varying driving forces of urban land expansion in China: insights from a spatial-temporal analysis. Sci Total Environ. (2021) 766:142591. 10.1016/j.scitotenv.2020.142591 33601670

[44] Wu X Hu S Kwaku AB Li Q Luo K Zhou Y. Spatio-temporal clustering analysis and its determinants of hand, foot and mouth disease in Hunan, China, 2009-2015. BMC Infect Dis. (2017) 17:645. 10.1186/s12879-017-2742-9 28946852 PMC5613322

[45] Wei JH Wu R Xing DX Shu J Hu SX Qin JB. Epidemiological characteristics and spatial epidemiology of hand-foot-mouth disease in Hunan Province, China, from 2008 to 2019. Zhongguo Dang Dai Er Ke Za Zhi. (2021) 23:1141–8. 10.7499/j.issn.1008-8830.2107031 34753546 PMC8580032

[46] Li T Yang Z Di B Wang M. Hand-foot-and-mouth disease and weather factors in Guangzhou, southern China. Epidemiol Infect. (2014) 142:1741–50. 10.1017/S0950268813002938 24267476 PMC9151230

[47] Bell ML O'Neill MS Ranjit N Borja-Aburto VH Cifuentes LA Gouveia NC. Vulnerability to heat-related mortality in Latin America: a case-crossover study in Sao Paulo, Brazil, Santiago, Chile and Mexico City, Mexico. Int J Epidemiol. (2008) 37:796–804. 10.1093/ije/dyn094 18511489 PMC2734062

[48] Kramer A Schwebke I Kampf G. How long do nosocomial pathogens persist on inanimate surfaces? A systematic review. BMC Infect Dis. (2006) 6:130. 10.1186/1471-2334-6-130 16914034 PMC1564025

[49] Onozuka D Hashizume M. The influence of temperature and humidity on the incidence of hand, foot, and mouth disease in Japan. Sci Total Environ. (2011) 4101:119–25. 10.1016/j.scitotenv.2011.09.055 22014509

[50] Qi H Chen Y Xu D Su H Zhan L Xu Z. Impact of meteorological factors on the incidence of childhood hand, foot, and mouth disease (HFMD) analyzed by DLNMs-based time series approach. Infect Dis Poverty. (2018) 7:7. 10.1186/s40249-018-0388-5 29391070 PMC5796399

[51] Ma E Lam T Wong C Chuang SK. Is hand, foot and mouth disease associated with meteorological parameters? Epidemiol Infect. (2010) 138:1779–88. 10.1017/S0950268810002256 20875200

[52] World Health Organization. WHO guidelines approved by the guidelines review committee. In:Atkinson J Chartier Y Pessoa-Silva CL Jensen P Li Y SetoWH, editors. Natural Ventilation for Infection Control in Health-Care Settings. Geneva: World Health Organization Copyright 2009 (2009). p. 18. 23762969

[53] He X Dong S Li L Liu X Wu Y Zhang Z. Using a Bayesian spatiotemporal model to identify the influencing factors and high-risk areas of hand, foot and mouth disease (HFMD) in Shenzhen. PLoS Negl Trop Dis. (2020) 14:e0008085. 10.1371/journal.pntd.0008085 32196496 PMC7112242

[54] Li L Qiu W Xu C Wang J A. spatiotemporal mixed model to assess the influence of environmental and socioeconomic factors on the incidence of hand, foot and mouth disease. BMC Public Health. (2018) 18:274. 10.1186/s12889-018-5169-3 29463224 PMC5819665

